# The Association Between Facial Width-to-Height Ratio (fWHR) and Sporting Performances: Evidence From Professional Basketball Players in Japan

**DOI:** 10.3389/fpsyg.2021.714819

**Published:** 2021-08-18

**Authors:** Shintaro Sato, Keita Kinoshita, Koichi Sekino, Haruka Amano, Yoshifumi Bizen, Hirotaka Matsuoka

**Affiliations:** ^1^Faculty of Sport Sciences, Waseda University, Tokyo, Japan; ^2^Graduate School of Sport Sciences, Waseda University, Tokyo, Japan; ^3^Faculty of Human Development, Kokugakuin University, Kanagawa, Japan

**Keywords:** facial structure, fWHR, achievement drive, aggression, athletic performance

## Abstract

Previous research in evolutionary psychology has highlighted the potential role of facial structures in explaining human behavior. The facial width-to-height ratio (fWHR) was found to be associated with testosterone-driven behavioral tendencies like achievement drive, aggression, and sporting success. The current study aimed to replicate such relationships using real-world data (i.e., professional basketball players; *N* = 482). Achievement drive, aggression, and sporting success were operationalized as field-goal attempts (FGA), the number of fouls committed (Foul), and player performance rating (EFF), respectively. The results indicated that fWHR was significantly associated with FGA and EFF, controlling for minutes of play and body-mass-index. The same results were obtained for separate analyses focusing on outsider players. However, analyses of inside players demonstrated that fWHR was associated only with EFF. The current research further provides empirical evidence supporting the effects of fWHR on achievement drive and sporting successes, although the effect sizes are notably small.

## Introduction

Faces play an important role in society as individuals communicate with facial expressions and draw inferences about others' personalities and behavioral tendencies based on their faces (Zebrowitz, 2018). Although such inferences are often inaccurate and undesirable, various research has highlighted that faces could be a predictor of human traits and behavior (Wong et al., 2011; Short et al., 2012). In this research stream, facial width-to-height ratio (fWHR; calculated by dividing the length between the left and right zygomatic arches by the length between the top of the lip and the bottom of the eyebrows) has commonly been utilized to better understand the relationships between facial characteristics and various outcomes (Geniole et al., 2015). For example, fWHR is positively associated with achievement drive (Lewis et al., 2012), psychopathic personality traits (Geniole et al., 2015; Anderl et al., 2016), and anti-social behavior (Stirrat and Perrett, 2010). The common explanation underlying such relationships is related to the testosterone level (Lefevre et al., 2013). It has been theorized that increased testosterone exposure during puberty can contribute to forming wider faces—greater fWHR, which in turn leads to aggressive traits and behavior (Marečková et al., 2011; Lefevre et al., 2013; Haselhuhn et al., 2015).

Based on the testosterone hypothesis explained above, fWHR could be a useful tool in certain contexts—Sport. Sporting success can be associated with testosterone-driven attributes (e.g., achievement drive, aggression; Tamiya et al., 2012; Tsujimura and Banissy, 2013). Indeed, previous literature highlighted the associations between fWHR and testosterone-driven attributes in sport (Carré and McCormick, 2008). Carré and McCormick (2008) found that fWHR is predictive of ice hockey penalties and aggressive behavior in a laboratory setting. Similarly, fWHR is associated with aggressive behavior (measured by the number of penalty cards received and fouls committed) among football players (Welker et al., 2015; Fujii et al., 2016). Trebický et al. (2014) demonstrated that fWHR is significantly associated with fighting success among mixed martial arts (MMA) athletes. Tsujimura and Banissy (2013) demonstrated that Japanese professional baseball players' fWHR is associated with their home run performance in two consecutive seasons. Although various scholars operationalized outcome variables differently, it seems warranted that fWHR is associated with sporting performances backed by achievement drive, aggression, and actual performances.

Nevertheless, previous literature has also reported inconsistent findings regarding fWHR (Haselhuhn et al., 2015; Kramer, 2015; Kosinski, 2017; Wang et al., 2019). For example, a large-scale study conducted by Kosinski (2017) found that fWHR was not associated with self-reported behavioral tendencies such as cooperativeness, impulsiveness, and impression management. Wang et al. (2019) indicated little evidence regarding the relationship between fWHR and anti-social behavioral tendencies. Similarly, some studies focused on athletes' fWHR have provided no support for the association between fWHR and testosterone-related outcomes. Kramer's (2015) study showed that commonwealth game athletes who compete in contact sports (e.g., boxing, judo) have greater fWHR than those who compete in non-contact sports (e.g., badminton, swimming), but the effect was negated when controlling for body-mass-index (BMI).

The replicability of published fWHR findings has recently been a primary concern.

Although some meta-analyses indicated significant results of fWHR (e.g., Haselhuhn et al., 2015; Giacomin and Rule, 2020), studies that obtained null findings have commonly used real-world small sample data (Wang et al., 2019). Hence, it is essential to further examine the robustness of the associations between fWHR and sporting performances by using real-world data with relatively large observations.

Accordingly, the current study assessed the relationship between fWHR and sporting performances by using actual professional basketball players who compete in Japan Professional Basketball League (B-League). In accordance with previous literature, we focus on (1) achievement drive, (2) aggression, and (3) sporting successes as outcome variables. Achievement drive has been defined as a mentality that encourages individuals to stand out in competition (Singh, 2011). Athletes who strongly pursue achievement can demonstrate certain behaviors that help them stand out from the crowd. In the context of basketball, scoring is one of the most crucial indicators that can influence the level of fame that athletes may receive. In fact, Berri et al. (2011) found that the scoring record can predict the draft order in NBA. Since achievement drive itself cannot guarantee that such behaviors end up successes, the current study operationalized achievement drive as field-goal-attempts (FGA). Aggression refers to actions that are intended to harm other people to achieve their results (Husman and Silva, 1984). The construct of aggression has been operationalized as the number of penalties received (e.g., fouls, yellow/red cards received, penalty minutes; Carré and McCormick, 2008; Goetz et al., 2013; Welker et al., 2015; Fujii et al., 2016). The current study also follows the previous literature and operationalized aggression as the number of fouls (Foul). Lastly, sporting successes can be viewed from various perspectives. However, previous literature has commonly focused on on-field performances of athletes or teams (Beedie et al., 2000; Wicker et al., 2012). Considering that the current study aimed to investigate the relationship between fWHR and sporting successes at the individual athlete level, the authors operationalized sporting success using player efficiency ratings (EFF). The current research can further provide evidence regarding the associations between fWHR and behavioral tendencies explained above in sport by utilizing real-world data.

## Methods

### Materials

To measure fWHR, photographs were obtained *via* the official player directory book for the 2019–2020 season published by B-League. After confirming that all the player images are forward-facing, two research assistants independently measured vertical and horizontal lengths by following the established approach (Weston et al., 2007; Özener, 2012; Lefevre et al., 2013). Specifically, we measured the vertical lengths between the highest point of the upper lip to brow. Face width was measured based on the horizontal distance from the left to the right zygomatic arch.

With regard to dependent measures, we obtained each player's performance statistics (i.e., FGA, Foul, and EFF) published by each team's official web pages. FGA and Foul are the total number of shots attempted and fouls committed throughout the season. EFF was calculated using the formula:

$$\begin{array}{l} \begin{array}{l} \text{EFF = }\left(\text{Points + Rebounds + Assists + Steals + Blocks}\right)\\ \;-\;(\text{Missed Field Goals + Missed Free Throws}\\ \text{ + Turnovers})\text{/the number of games played}. \end{array} \end{array}$$

It is common to use EFF to understand the contribution of players to a game in the National Basketball Association (NBA). EFF has been frequently used as a performance measurement of basketball players in previous empirical studies (e.g., Staunton et al., 2017; Kingsley et al., 2021). Ninety players were excluded from the further analyses due to unavailability of performance data. Twenty seven players who competed in <10 games were excluded from further analyses (Tsujimura and Banissy, 2013), leaving 482 observations.

It is also important to control variables that can potentially influence the association between fWHR and sporting performance. Based on previous literature, we controlled minutes of play (Krenn and Meier, 2018) and BMI (Mayew, 2013; Kramer, 2015; Fujii et al., 2016). Krenn and Meier (2018) suggested that sporting successes can be strongly influenced by each player's playing time. Their study found no evidence of fWHR when controlling for minutes of gameplay. For the same reason, we also controlled BMI. Deaner et al. (2012) found that the effect of fWHR on aggression among ice hockey players was canceled when controlling for body weight. The official webpage of B-league makes players' minutes of play, height, and weight data available, being calculated to develop BMI data for each player.

### Statistical Analyses

We first ran descriptive analyses to assess mean, standard deviation, minimum and maximum scores for all variables included in this study. Pearson correlation was then employed to evaluate inter-correlations among variables. We then performed a series of hierarchical regression models to investigate the associations between fWHR and professional basketball players' performance. Specifically, the first step included minutes of play and BMI in the model as control variables. In the second step, we included fWHR as a predictor variable. The effect size was interpreted based on the *R*^2^ changes.

After the omnibus analyses above, we also conducted separate analyses for outside versus inside players based on previous literature, suggesting that athletes' playing positions can influence their performance statistics (Welker et al., 2015; Fujii et al., 2016; Krenn and Meier, 2018). Indeed, a study conducted by Ferioli et al. (2018) also demonstrated that basketball players' physical profile is heterogeneous depending on playing positions. Players' positions were determined based on the information available on the official B-league webpage. Specifically, we categorized point guard (PG), shooting guard (SG), and small forward (SF) as outside players, whereas power forward (PF) and center (C) as inside players.

## Results

**Figure 1:**
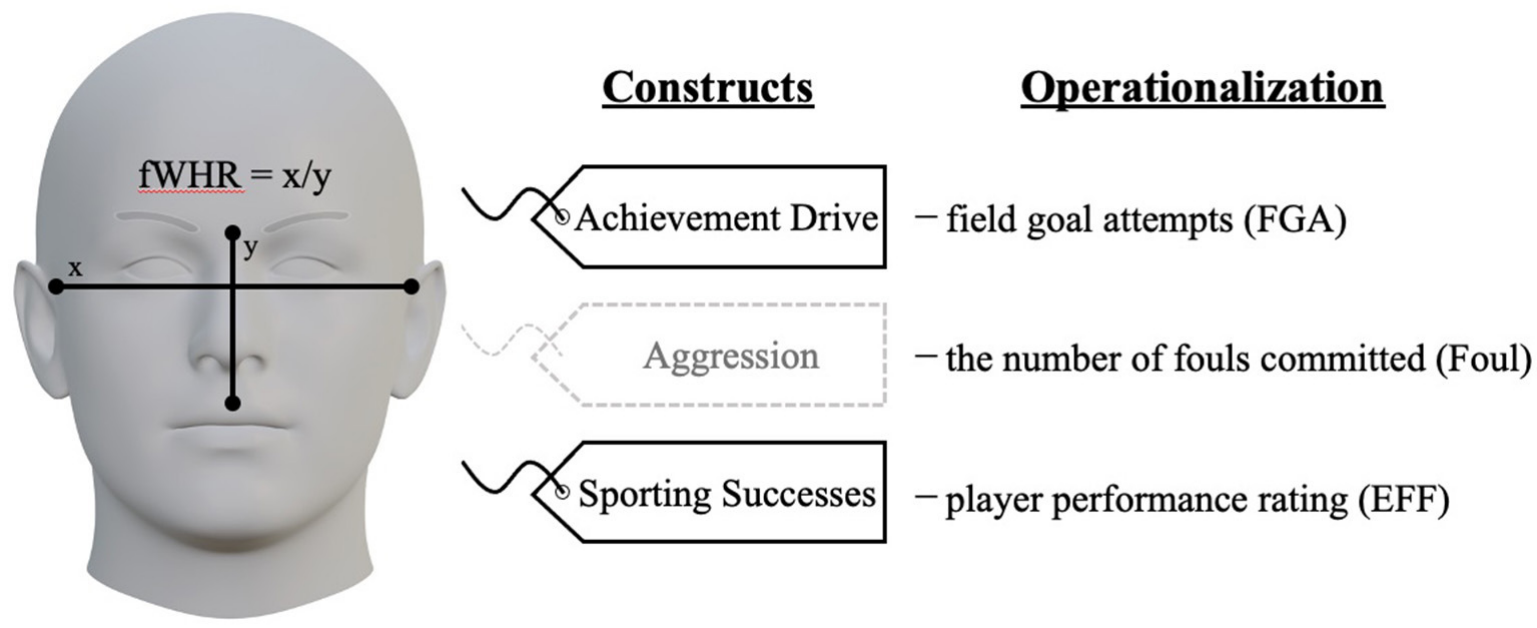
The summary of the results.

Descriptive analyses indicated that players' minutes of play ranged between 17.70 and 1780.82 minutes, with an average of 685.53 (*SD* = 385.79). BMI ranged from 20.45 to 32.03 with a mean of 24.59 (*SD* = 1.65). With regard to the dependent measure, fWHR scores in this study ranged from 1.56 to 2.67, with a mean of 1.96 (*SD* = 0.18). It is important to note that the inter-coder consistency of fWHR was 97.2%, ensuring the reliability of fWHR data in this study (Deaner et al., 2012). FGA fell between 3 and 1030 with a mean of 217.91 (*SD* = 166.55). Regarding Foul, the score ranged from 0 to 135, whose average score was 60.86 (*SD* = 29.54). EFF scores were in the range of 0 to 33, with an average of 8.39 (*SD* = 7.57). The official webpage of the league also published players' age, which ranged from 20 to 49 years old, and the mean was 28.42. Descriptive statistics and correlations among variables are shown in Tables 1, 2. The visual summary of the results can be found in Figure 1.

**Table 1:** Descriptive statistics.

		**All players (** ***n*** **=** **482)**				**Outside players (** ***n*** **=** **318)**				**Inside players (** ***n*** **=** **164)**			
		***M***	***SD***	**Min**	**Max**	***M***	***SD***	**Min**	**Max**	***M***	***SD***	**Min**	**Max**
1	Minutes of Play	685.53	385.79	17.70	1780.82	664.78	353.30	23.55	1737.73	725.76	440.43	17.70	1780.82
2	BMI	24.59	1.65	20.45	32.03	24.02	1.25	20.45	29.19	25.70	1.78	21.04	32.03
3	fWHR	1.96	0.18	1.56	2.67	1.94	0.16	1.56	0.67	2.00	0.21	1.56	2.50
4	FGA	217.91	166.55	3.00	1030.00	195.07	134.65	9.00	904.00	262.21	208.80	3.00	1030.00
5	Foul	60.86	29.54	0.00	135.00	59.18	29.03	0.00	133.00	64.12	30.33	0.00	135.00
6	EFF	8.39	7.57	0.00	33.00	5.86	4.36	0.00	33.00	13.29	9.77	1.00	32.00

*BMI, body mass index; fWHR, facial width-to-height ratio; FGA, field goal attempt; Foul, number of fouls committed; EFF, performance efficiency rating*.

**Table 2:** Correlations.

		**1**	**2**	**3**	**4**	**5**
1	Minutes of play	–				
2	BMI	0.14^**^	–			
3	fWHR	0.07	0.23^**^	–		
4	FGA (field goal attempt)	0.90^**^	0.12^**^	0.20^**^	–	
5	Foul (number of fouls committed)	0.80^**^	0.02	0.11^*^	0.64^**^	–
6	EFF (performance efficiency rating)	0.68^**^	0.25^**^	0.33^**^	0.81^**^	0.45^**^

* *p < 0.05*.

** *p < 0.01*.

The results of the regression analyses are represented in Table 3. In the first step, minutes of play and BMI explained 80.7, 63.4, and 52.2% of variances in FGA, Foul, and EFF of overall players. In Step 2, fWHR significantly predicted FGA (*B* = 39.06, 95% CIs [2.11, 76.01], *p* < 0.05, β = 0.04, Δ*R*^2^ = 0.002) and EFF (*B* = 6.37, 95% CIs [3.78, 8.97], *p* < 0.001, β = 0.15, Δ*R*^2^ = 0.02). In addition to the minutes of play and BMI, fWHR explained 0.2 and 2.2 % of the variances in FGA and EFF, respectively. However, we did not find evidence in Foul (*B* = −6.70, 95% CIs [−15.73, 2.33], *p* = 0.15, β = −0.04, Δ*R*^2^ =0.002).

**Table 3:** Results of regression analyses for FGA, Foul, and EFF.

	**FGA**					**Foul**					**Efficiency**				
	***B***	**95% CI**	**β**	**Δ*R*^**2**^**	***p***	***B***	**95% CI**	**β**	**Δ*R*^**2**^**	***p***	***B***	**95% CI**	**β**	**Δ*R*^**2**^**	***p***
**All players (** ***n*** **=** **482)**
Step 1				0.807***	<0.001				0.634***	<0.001				0.522***	<0.001
Minutes of play	0.38***	[0.36, 0.40]	0.88***		<0.001	0.06***	[0.06, 0.07]	0.80***		<0.001	0.01***	[0.011,0.014]	0.65***		<0.001
BMI	7.98***	[3.96, 11.99]	0.08***		<0.001	−0.03	[−1.01, 0.95]	−0.01		0.95	1.13***	[0.84, 1.41]	0.25***		<0.001
Step 2				0.002*	<0.05				0.002	0.15				0.022***	<0.001
Minutes of play	0.38***	[0.36, 0.40]	0.88***		<0.001	0.06***	[0.06, 0.07]	0.80***		<0.001	0.01***	[0.011,0.014]	0.64***		<0.001
BMI	7.01***	[2.91, 11.12]	0.07***		<0.001	0.13	[−0.87, 1.14]	0.01		0.80	0.97***	[0.68, 1.26]	0.21***		<0.001
FWHR	39.06*	[2.11, 76.01]	0.04*		<0.05	−6.699	[−15.73, 2.33]	−0.04		0.15	6.37***	[3.78, 8.97]	0.15***		<0.001
**Outside players (** ***n*** **=** **318)**
Step 1				0.799***	<0.001				0.631***	<0.001				0.562***	<0.001
Minutes of play	0.341***	[0.32, 0.36]	0.89***		<0.001	0.07***	[0.06, 0.07]	0.80***		<0.001	0.01***	[0.008, 0.01]	0.75***		<0.001
BMI	−0.13	[−5.49, 5.24]	−0.001		0.963	−0.13	[−1.70, 1.43]	−0.01		0.87	0.07	[−0.16, 0.35]	0.03		0.46
Step 2				0.006**	<0.01				0.002	0.16				0.031***	<0.001
Minutes of play	0.34***	[0.32, 0.36]	0.90***		<0.001	0.07***	[0.06, 0.07]	0.79***		<0.001	0.01***	[0.008, 0.10]	0.75***		<0.001
BMI	−1.49	[−6.85, 3.86]	−0.01		0.584	0.04	[−1.54, 1.63]	0.01		0.96	−0.01	[−0.25, 0.25]	−0.01		0.99
FWHR	65.35**	[24.79, 105.91]	0.08**		<0.01	−8.55	[−20.55, 3.44]	−0.05		0.16	4.71***	[2.81, 6.61]	0.18***		<0.001
**Inside players (** ***n*** **=** **164)**
Step 1				0.839	<0.001				0.647	<0.001				0.597***	<0.001
Minutes of play	0.43***	[0.40, 0.46]	0.91***		<0.001	0.06***	[0.05, 0.06]	0.81***		<0.001	0.02***	[0.015, 0.019]	0.76***		<0.001
BMI	2.83	[−4.66, 10.32]	0.02		0.46	−0.14	[−1.76, 1.47]	−0.01		0.86	0.25	[−0.31, 0.80]	0.05		0.38
Step 2				0.001	0.67				0.001	0.63				0.017**	<0.01
Minutes of play	0.43***	[0.40, 0.46]	0.91***		<0.001	0.06***	[0.05, 0.06]	0.81***		<0.001	0.02***	[0.014, 0.019]	0.75***		<0.001
BMI	3.15	[−4.50, 10.80]	0.03		0.42	−0.06	[−1.71, 1.58]	−0.01		0.94	0.10	[−0.45, 0.66]	0.02		0.72
FWHR	−13.79	[−78.37, 50.78]	−0.01		0.67	−3.45	[−17.35, 10.45]	−0.02		0.63	6.29**	[1.61, 10.97]	0.13**		<0.01

With regard to the position-based separate analyses, minutes of play and BMI explained 79.9, 63.1, and 56.2% of variances in FGA, Foul, and EFF of outside players in step 1. In Step 2, the results showed that fWHR was positively associated with FGA (*B* = 65.35, 95% CIs [24.79, 105.91], *p* < 0.01, β = 0.08, Δ*R*^2^ = 0.006) and EFF (*B* = 4.71, 95% CIs [2.81, 6.61], *p* < 0.001, β = 0.18, Δ*R*^2^ = 0.03) in the outside players group while Foul was not predicted by fWHR (*B* = −8.55, 95% CIs [−20.55, 3.44], *p* = 0.16, β = −0.05, Δ*R*^2^ =0.002). Thus, fWHR significantly explained 0.6 and 3.1 % of the variances in FGA and EFF, respectively. Lastly, we found that minutes of play and BMI explained 83.9, 64.7, and 59.7% of variances in FGA, Foul, and EFF of inside players. Only EFF (*B* = 6.29, 95% CIs [1.61, 10.97], *p* < 0.01, β = 0.13, Δ*R*^2^ = 0.02) was significantly predicted by fWHR while no evidence was shown in FGA (*B* = −13.79, 95% CIs [−78.37, 50.78], *p* = 0.67, β = −0.01, Δ*R*^2^ =0.001) and Foul (*B* = 3.45, 95% CIs [−17.35, 10.45], *p* = 0.63, β = −0.02, Δ*R*^2^ =0.001) in the inside player group. The *R*^2^ change for EFF of inside players was 1.7%.

## Discussion

Previous literature has provided mixed findings regarding the association of fWHR on sporting performances (Carré and McCormick, 2008; Deaner et al., 2012; Tsujimura and Banissy, 2013; Kramer, 2015; Welker et al., 2015; Fujii et al., 2016). The present study was conducted to investigate the relationship between fWHR and testosterone-driven outcomes such as professional basketball players' achievement drive (i.e., FGA), aggression (i.e., Foul), and sporting successes (i.e., EFF) by utilizing real-world data (i.e., professional basketball players). The results indicated that fWHR was significantly related to FGA and EFF in the total samples, although the effect size was trivial. Such findings were consistent for the group of outside players, whereas fWHR only predicted EFF for the inside player group. Inconsistent with the previous literature, the association between fWHR and aggression was not supported. Overall, it is concluded that the testosterone hypothesis was partially supported.

There are several theoretical implications from the above findings. First, fWHR could be considered a meaningful predictor of achievement drive (β = 0.04, 95% CIs [2.11, 76.01], *p* < 0.05, Δ*R*^2^ = 0.002) and sporting successes (β = 0.15, 95% CIs [3.78, 8.97], *p* < 0.001) in professional basketball in Japan. Based on the testosterone hypothesis, scholars found that individuals with large fWHR are more competitive and successful (Wong et al., 2011; Lewis et al., 2012; Stirrat and Perrett, 2012). Previous literature focused on sport performances also yielded similar findings. For example, fWHR is significantly associated with MMA athletes' fighting successes (Trebický et al., 2014), baseball players' home runs (Tsujimura and Banissy, 2013), and football players' goals and assists (Welker et al., 2015). Nevertheless, some empirical studies demonstrated very little or null effects of fWHR (Haselhuhn et al., 2015; Kramer, 2015; Kosinski, 2017; Wang et al., 2019). In particular, the refuting evidence has been reported when controlling for various characteristics of players (e.g., BMI, minutes of play; Mayew, 2013; Kramer, 2015; Krenn and Meier, 2018). In this sense, since we also controlled for BMI and minutes of play, it deemed acceptable to conclude that the effect of fWHR on achievement drive as well as performance in basketball players is—while small—replicated.

Second, the relationship between fWHR and aggression did not turn out statistically significant (β = −0.04, 95% CIs [−15.73, 2.33], *p* = 0.15). The results of the current study were somewhat inconsistent because the relationship between fWHR and aggression has been relatively oft-supported with various operationalizations (e.g., fouls, yellow/red cards received, penalty minutes; Carré and McCormick, 2008; Goetz et al., 2013; Welker et al., 2015; Fujii et al., 2016) and even in meta-analytic projects (Geniole et al., 2015; Haselhuhn et al., 2015). However, some prior studies have also reported the null findings of fWHR on aggression (e.g., Deaner et al., 2012; Kramer, 2015; Krenn and Meier, 2018). Moreover, it is imperative to note that the supporting evidence reported in previous literature was small in effect sizes with somewhat inconsistent findings when separate analyses based on players' positions were conducted (e.g., Fujii et al., 2016). In this sense, the current research further added to the empirical evidence refuting the effect of fWHR on aggression. A possible interpretation for this non-significant finding is our operationalization of aggression. Based on previous literature, fouls could be an appropriate variable that operationalizes aggression. Nevertheless, committing fouls in basketball is highly strategic (Ángel et al., 2006). Hence, players might commit fouls outside of the influence of aggression. Although FGA, Foul, and EFF could be appropriate operationalizations in basketball among publicly available data, it would have been more desirable to obtain more detailed player statistics. One example could be the number of technical or unsportsmanlike fouls, which could be linked more to aggressive traits.

The practical implication that the current study can highlight can be related to the player selection. In basketball, team performance is dependent upon a variety of qualities. Therefore, coaches and sport scientists need to understand the complex player selection dynamics (Balli and Korukoglu, 2014). Consistent with the association between BMI and performances observed in this study, Drinkwater et al. (2008) also emphasized the importance of the size of basketball players. However, it can be challenging to secure a “big-man” even in the professional basketball market. In such situations, incorporating the information about human face structure may contribute to effective player selection.

There were several research limitations in this study. The sample representation and generalizability were the first limitations due to the highly selective sample (i.e., professional basketball players in Japan). It could be possible to include fWHR data of non-professional athletes (e.g., college athletes) from various sports and test the relationship with standardized performance data. By doing so, concerns regarding alternative explanations about the restricted sample and a particular sport would be minimized. Second, although we attempted to investigate the association between fWHR and performances based on players' positions as a potential moderator. Future research should also consider other moderating variables that can alter the relationship between fWHR and focal variables. For example, Goetz et al. (2013) found that social status moderated the relationship between fWHR and aggression with the sample of NHL players. Specifically, fWHR gives a meaningful impact on aggression when the target individuals are low in social status. Social status can be operationalized as players' salaries in sport (Goetz et al., 2013). However, we could not incorporate it into the current study due to data availability issues.

In conclusion, the current research provided valuable additions to the literature. Expressly, this study further provided supporting evidence regarding the relationship between basketball players' fWHR and achievement drive as well as sporting successes. Nevertheless, the relationship between fWHR and aggression should be re-considered. Considering the above limitations, scholars should exercise caution in generalizing the findings. Meanwhile, we welcome future scholarly efforts in extending our research by incorporating various moderators, which will contribute to the growing body of evolutionary psychology literature that focuses on physical characteristics and sporting performances.

## Data Availability Statement

The original contributions presented in the study are included in the article/Supplementary Material, further inquiries can be directed to the corresponding author.

## Author Contributions

SS: conceptualization, data collection, data analyses, and original draft writing. KK: manuscript writing/editing and data analyses. KS, HA, and YB: data collection. HM: reviewing manuscript and supervision. All authors contributed to the article and approved the submitted version.

## Conflict of Interest

The authors declare that the research was conducted in the absence of any commercial or financial relationships that could be construed as a potential conflict of interest.

## Publisher's Note

All claims expressed in this article are solely those of the authors and do not necessarily represent those of their affiliated organizations, or those of the publisher, the editors and the reviewers. Any product that may be evaluated in this article, or claim that may be made by its manufacturer, is not guaranteed or endorsed by the publisher.

## References

[B1] Anderl C. Hahn T. Schmidt A. K. Moldenhauer H. Notebaert K. Clément C. C.. (2016). Facial width-to-height ratio predicts psychopathic traits in males. Person. Individ. Differ. 88, 99–101. 10.1016/j.paid.2015.08.057

[B2] Ángel G. M. Evangelos T. Alberto L. (2006). Defensive systems in basketball ball possessions. Int. J. Perform. Anal. Sport 6, 98–107. 10.1080/24748668.2006.11868358

[B3] Balli S. Korukoglu S. (2014). Development of a fuzzy decision support framework for complex multi-attribute decision problems: a case study for selecting skillful basketball players. Exp. Syst. 31, 56–69. 10.1111/exsy.12002

[B4] Beedie C. J. Terry P. C. Lane A. M. (2000). The profile of mood states and athletic performance: two meta-analyses. J. Appl. Sport Psychol. 12, 49–68. 10.1080/10413200008404213

[B5] Berri D. J. Brook S. L. Fenn A. J. (2011). From college to the pros: predicting the NBA amateur player draft. J. Prod. Anal. 35, 25–35. 10.1007/s11123-010-0187-x

[B6] Carré J. M. McCormick C. M. (2008). In your face: facial metrics predict aggressive behaviour in the laboratory and in varsity and professional hockey players. Proc. R. Soc. B: Biol. Sci. 275, 2651–2656. 10.1098/rspb.2008.0873 18713717 PMC2570531

[B7] Deaner R. O. Goetz S. M. M. Shattuck K. Schnotala T. (2012). Body weight, not facial width-to-height ratio, predicts aggression in pro hockey players. J. Res. Pers. 46, 235–238. 10.1016/j.jrp.2012.01.005

[B8] Drinkwater E. J. Pyne D. B. McKenna M. J. (2008). Design and interpretation of anthropometric and fitness testing of basketball players. Sports Med. 38, 565–578. 10.2165/00007256-200838070-00004 18557659

[B9] Ferioli D. Rampinini E. Bosio A. La Torre A. Azzolini M. Coutts A. J. (2018). The physical profile of adult male basketball players: differences between competitive levels and playing positions. J. Sports Sci. 36, 2567–2574. 10.1080/02640414.2018.1469241 29697296

[B10] Fujii T. Goto A. Takagishi H. (2016). Does facial width-to-height ratio predict Japanese professional football players' athletic performance? Lett. Evol. Behav. Sci. 7, 37–40. 10.5178/lebs.2016.49

[B11] Geniole S. N. Denson T. F. Dixson B. J. Carré J. M. McCormick C. M. (2015). Evidence from meta-analyses of the facial width-to-height ratio as an evolved cue of threat. PLoS ONE 10:e0132726. 10.1371/journal.pone.0132726 26181579 PMC4504483

[B12] Giacomin M. Rule N. O. (2020). How static facial cues relate to real-world leaders' success: a review and meta-analysis. Eur. Rev. Soc. Psychol. 31, 120–148. 10.1080/10463283.2020.1771935

[B13] Goetz S. M. Shattuck K. S. Miller R. M. Campbell J. A. Lozoya E. Weisfeld G. E.. (2013). Social status moderates the relationship between facial structure and aggression. Psychol. Sci. 24, 2329–2334. 10.1177/0956797613493294 24068116

[B14] Haselhuhn M. P. Ormiston M. E. Wong E. M. (2015). Men's facial width-to-height ratio predicts aggression: a meta-analysis. PLoS ONE 10:e0122637. 10.1371/journal.pone.0122637 25849992 PMC4388848

[B15] Husman B. F. Silva J. M. (1984). Aggression in sport: definitional and theoretical considerations. Psychological Foundations of Sport, 246–260. Champaign, IL: Human Kinetics.

[B16] Kingsley N. Amsbaugh S. Papadakis Z. Morgan G. Boolani A. (2021). Sex moderates the fitness tests-performance index relationship in collegiate basketball: a case study. Int. J. Exerc. Sci. Conf. Proc. 2, 17.

[B17] Kosinski M. (2017). Facial width-to-height ratio does not predict self-reported behavioral tendencies. Psychol. Sci. 28, 1675–1682. 10.1177/0956797617716929 28976810

[B18] KramerR. S. S. (2015). Facial width-to-height ratio in a large sample of commonwealth games athletes. Evol. Psychol. 13, 197–209. 10.1177/14747049150130011225714799

[B19] Krenn B. Meier J. (2018). Does facial width-to-height ratio predict aggressive behavior in association football? Evol. Psychol. 16, 1–8. 10.1177/1474704918818590

[B20] Lefevre C. E. Lewis G. J. Perrett D. I. Penke L. (2013). Telling facial metrics: facial width is associated with testosterone levels in men. Evol. Human Behav. 34, 273–279. 10.1016/j.evolhumbehav.2013.03.005

[B21] Lewis G. J. Lefevre C. E. Bates T. C. (2012). Facial width-to-height ratio predicts achievement drive in US presidents. Pers. Individ. Differ. 52, 855–857. 10.1016/j.paid.2011.12.030

[B22] Marečková K. Weinbrand Z. Chakravarty M. M. Lawrence C. Aleong R. Leonard G. Paus T. (2011). Testosterone-mediated sex differences in the face shape during adolescence: subjective impressions and objective features. Hormones Behav. 60, 681–690. 10.1016/j.yhbeh.2011.09.004 21983236

[B23] Mayew W. J. (2013). Reassessing the association between facial structure and baseball performance: a comment on Tsujimura and Banissy. Biol. Lett. 9:20130538. 10.1098/rsbl.2013.0538 24026348 PMC3971694

[B24] Özener B. (2012). Facial width-to-height ratio in a Turkish population is not sexually dimorphic and is unrelated to aggressive behavior. Evol. Human Behav. 33, 169–173. 10.1016/j.evolhumbehav.2011.08.001

[B25] Short L. A. Mondloch C. J. McCormick C. M. Carré J. M. Ma R. Fu G.. (2012). Detection of propensity for aggression based on facial structure irrespective of face race. Evol. Human Behav. 33, 121–129. 10.1016/j.evolhumbehav.2011.07.002 22611331 PMC3352668

[B26] Singh K. (2011). Study of achievement motivation in relation to academic achievement of students. Int. J. Educ. Plan. Admin. 1, 161–171.

[B27] Staunton C. Gordon B. Custovic E. Stanger J. Kingsley M. (2017). Sleep patterns and match performance in elite Australian basketball athletes. J. Sci. Med. Sport 20, 786–789. 10.1016/j.jsams.2016.11.016 28169152

[B28] Stirrat M. Perrett D. I. (2010). Valid facial cues to cooperation and trust: male facial width and trustworthiness. Psychol. Sci. 21, 349–354. 10.1177/0956797610362647 20424067

[B29] Stirrat M. Perrett D. I. (2012). Face structure predicts cooperation: men with wider faces are more generous to their in-group when out-group competition is salient. Psychol. Sci. 23, 718–722. 10.1177/0956797611435133 22623509

[B30] Tamiya R. Lee S. Y. Ohtake F. (2012). Second to fourth digit ratio and the sporting success of sumo wrestlers. Evol. Human Behav. 33, 130–136. 10.1016/j.evolhumbehav.2011.07.003

[B31] Trebický V. Fialová J. Kleisner K. Roberts S. C. Little A. C. Havlíček J.. (2014). Further evidence for links between facial width-to-height ratio and fighting success: commentary on Zilioli et al. (2014). Aggress. Behav. 41, 331–334. 10.1002/ab.21559 25236530

[B32] Tsujimura H. Banissy M. J. (2013). Human face structure correlates with professional baseball performance: insights from professional Japanese baseball players. Biol. Lett. 9:20130140. 10.1098/rsbl.2013.0140 23576779 PMC3645049

[B33] Wang D. Nair K. Kouchaki M. Zajac E. J. Zhao X. (2019). A case of evolutionary mismatch? Why facial width-to-height ratio may not predict behavioral tendencies. Psychol. Sci. 30, 1074–1081. 10.1177/0956797619849928 31180794

[B34] Welker K. M. Goetz S. M. Galicia S. Liphardt J. Carré J. M. (2015). An examination of the associations between facial structure, aggressive behavior, and performance in the 2010 World Cup association football players. Adapt. Human Behav. Physiol. 1, 17–29. 10.1007/s40750-014-0003-3

[B35] Weston E. M. Friday A. E. Liò P. (2007). Biometric evidence that sexual selection has shaped the hominin face. PLoS ONE 2:e710. 10.1371/journal.pone.0000710 17684556 PMC1937021

[B36] Wicker P. Prinz J. von Hanau T. (2012). Estimating the value of national sporting success. Sport Manag. Rev. 15, 200–210. 10.1016/j.smr.2011.08.007

[B37] Wong E. M. Ormiston M. E. Haselhuhn M. P. (2011). A face only an investor could love: CEOs' facial structure predicts their firms' financial performance. Psychol. Sci. 22, 1478–1483. 10.1177/0956797611418838 22042727

[B38] Zebrowitz L. (2018). Reading Faces: Window to the Soul?. Milton Park: Routledge. 10.4324/9780429493188

